# Novel insights into the role of bisphenol A (BPA) in genomic instability

**DOI:** 10.1093/narcan/zcae038

**Published:** 2024-09-24

**Authors:** Anastasia Hale, George-Lucian Moldovan

**Affiliations:** Department of Biochemistry and Molecular Biology, The Pennsylvania State University College of Medicine, Hershey, PA 17033, USA; Department of Biochemistry and Molecular Biology, The Pennsylvania State University College of Medicine, Hershey, PA 17033, USA

## Abstract

Bisphenol A (BPA) is a phenolic chemical that has been used for over 50 years in the manufacturing of polycarbonate and polyvinyl chloride plastics, and it is one of the highest volume chemicals produced worldwide. Because BPA can bind to and activate estrogen receptors, studies have mainly focused on the effect of BPA in disrupting the human endocrine and reproductive systems. However, BPA also plays a role in promoting genomic instability and has been associated with initiating carcinogenesis. For example, it has been recently shown that exposure to BPA promotes the formation of single stranded DNA gaps, which may be associated with increased genomic instability. In this review, we outline the mechanisms by which BPA works to promote genomic instability including chromosomal instability, DNA adduct formation, ROS production, and estrogen receptor (ER) activation. Moreover, we define the ways in which BPA promotes both carcinogenesis and resistance to chemotherapy, and we provide critical insights into future directions and outstanding questions in the field.

## Graphical Abstract

**Graphical Abstract:**
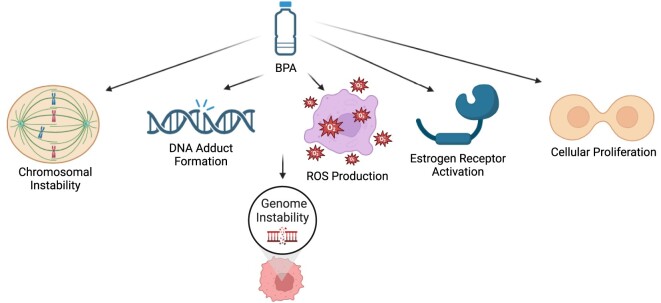

## Introduction

A phenolic chemical which has been used for over 50 years in the manufacturing of polycarbonate plastics, epoxy resins, thermal paper and polyvinyl chloride plastics, bisphenol-A (BPA) ranks in the top 2% of high-production-volume chemicals, with 5–6 billion pounds produced annually worldwide (1). BPA is an estrogenic compound which has been shown to bind to the nuclear estrogen receptor (ER) alpha and beta as well as the membrane-associated GPR30 (2,3). Because of its mass production, exposure to BPA is extremely prevalent. For example, a study in 2004 revealed that from a sample of 2517 individuals over the age of 6, 93% were found to have detectable levels of BPA in their urine (1). As of 2021, the median creatinine-adjusted urinary BPA concentration in adult humans was estimated to be 1.76 μg/g (95% PI: 0.79–2.73), and the pooled estimate for serum BPA was 1.75 μg/l (95% PI: 0–10.58) (4).

The main mechanism of exposure to BPA is through the diet, as BPA is capable of leaching through plastic products into proximal foods and liquids (5). BPA can also enter the body via inhalation of dust or fumes from burned plastics. Interestingly, populations with particularly high levels of BPA exposure include infants, especially those in neonatal intensive care units, and dialysis patients, due to leaching of BPA from medical equipment into fluids entering the body (6–8). Additionally, a 2014 study revealed that U.S. manufacturing workers in factories producing BPA containing products exhibited urinary BPA levels about 70 times greater than that of the general population, mainly via inhalation and dermal absorption (9).

Questions regarding the safety of BPA began to emerge around the year 2003, when a paper describing potential harm from BPA exposure was published (10). After accidental exposure to BPA from animal cages, the oocytes of female mice were shown to exhibit a sudden, spontaneous increase in meiotic disturbances, including aneuploidy (10). Since then, exposure to BPA has been associated with a wide variety of adverse health effects such as abnormalities in reproductive organ function, placental dysfunction, precocious puberty, and neurological impairment (11,12). For example, BPA exposure has been shown to upregulate the expression of gonadotropin releasing hormone (GnRH) and follicle stimulating hormone (FSH), hormones involved in the regulation of the menstrual cycle and spermatogenesis (13). In addition, BPA has also been shown to inhibit the synthesis of testosterone and increase the expression of aromatase and estrogen in mice pups (13).

Despite this evidence, the US Environmental Protection Agency (EPA) in conjunction with the Food and Drug Administration (FDA), the Centers for Disease Control and Prevention (CDC) and the National Institute of Environmental Health Sciences (NIEHS) have not taken regulatory action in regards to BPA, stating that these studies are insufficient for risk assessment due to either the dose used, flaws in some of the study designs, scientific uncertainty concerning the relevance to health and ecological hazard of the reported effects, and the inability of other researchers to reproduce the effects in standardized studies (14). In 2012, these organizations collaborated to form the Consortium Linking Academic and Regulatory Insights on BPA Toxicity (CLARITY-BPA) program with the goal of studying the full range of potential health effects from exposure to BPA. While BPA exposure increased the incidence of pathological lesions such as mammary gland adenocarcinoma and follicular ovarian cysts, the study concluded that these effects were not dose responsive, sometimes occurring in only one low or intermediate dose group and did not demonstrate a clear pattern of consistent responses within or across organs (15).

As of today, the EPA and FDA have established a safe reference dose (RfD) of BPA for humans at 50 μg/kg/day. This value is based on a 1982 study by the National Toxicology Program (NTP) that concluded that while pharmacological doses of BPA induce some cancers in both male and female adult rodents, it is not a robust carcinogen at doses relevant to human exposure (16). Important to consider however, is that BPA follows a non-monotonic dose-response curve, meaning that both low and high doses can produce similar phenotypes (17,18). While concentrations utilized in animal model experiments tend to hover around the RfD, cell culture experiments typically use a higher concentration of BPA, albeit for a much lesser period of time (Table 1). Whether higher levels of exposure for a shorter duration mimic the lifetime exposure in most humans is still unknown. Given that BPA, like most estrogenic chemicals, does not follow a typical linear dose response relationship, it is imperative to consider the relevance of these studies and how they may translate to human health.

**Table 1.** Summary of BPA doses and model systems used in cited studies

Study	Concentration of BPA found	Population
Colorado-Yohar *et al.*, 2021	Urine: 1.76 μg/g (95% PI: 0.79–2.73), Serum: 1.75 μg/l (95% PI: 0–10.58)	Adult humans (meta-analysis)
Calafat *et al.*, 2009	Urine: 1.6 to 946 μg/l (free mean = 30.3 μg/l)	Neonatal ICU premature infants
Kanno *et al.*, 2007	Serum: HD (5.3 ± 0.3 ng/ml) PD (3.8 ± 0.2 ng/ml)	Hemodialysis (HD) and peritoneal dialysis (PD) patients
Hines *et al.*, 2017	Urine: 88.0 μg/g (range 0.78–18900 μg/g)	US plastic manufacturing workers
**Study**	**Concentration of BPA used**	**Model used**
Hunt *et al.*, 2003	20, 40 or 100 ng/g body weight for 6–8 days	Female mice
Xi *et al.*, 2011	12, 25 and 50 mg/kg/day for 5 weeks	Six-week-old male and female CD-1 mice
Wang *et al.*, 2014	25 μg/kg/day from puberty – 3 weeks	Female Balb/c mice
Wadia *et al.*, 2013	250 ng/kg/day from E8 to parturition	Female C57BL/6-ERα± mice
Betancourt *et al.*, 2012	50–250 μg/kg/day from birth to PND21	Sprague–Dawley rats
Ayyanan *et al.*, 2011	0.6 μg–1.2 mg/kg/day from E1 to PND24	C57BL/6 mice
Allard and Colaiacovo, 2010	100 μM, 500 μM and 1 mM	*C. elegans*
Kim *et al.*, 2019	100 nM for 5 h	HeLa, MCF-7 and MDA-MB-231 cells
Atkinson and Roy, 1995	200 mg/kg, single IP injection	CD1 male rats
Zhao *et al.*, 2018	0.1 μM for 18 h	MCF-7 cell DNA
Izzotti *et al.*, 2009	200 mg/kg body weight for 8 days	CD-1 albino mice
Hu *et al.*, 2021	100 μM for 24 h	HEK 293T, IMR-90 and Hep G2 cells
Fic *et al.*, 2013	0.1 to 10 μM for 4 and 24 h	Hep G2 cells
Mokra *et al.*, 2016	0.01 to 10 μg/ml for 1 and 4 h	PBMCs
Hale *et al.*, 2023	200 μM for 2 h	HeLa, U2OS and RPE1 cells
Wang *et al.*, 2019	50 μg/kg/day for 10 weeks	3-week-old male CD-1 mice
Michałowicz *et al.*, 2015	0.06 to 500 μM for 1 h	PBMCs
Xin *et al.*, 2014	0, 25, 50 and 100 μM for 24 h	INS-1 cells
Bindhumol *et al.*, 2003	0.2, 2.0 and 20 μg/kg body weight per day for 30 days	Albino male rats of Wistar strain
Iso *et al.*, 2006	100 μM for 3–24 h	MCF-7 cells
Kabil at al., 2008	5 μM for 24 h	MCF-7 cells
Pfeifer *et al.*, 2015	10 and 100 nM for 24 h	MCF10A, 184A1, MCF-7 and MDA-MB-231 cells
Zhang *et al.*, 2022	20 mg/kg body weight/day for 30 weeks	3–4-week-old female SD rats
Pupo *et al.*, 2012	1 μM BPA for 48 h	SKBR3 cells and CAFs
Dairkee *et al.*, 2012	100 nM BPA for 7 days	HRBECs
Deb *et al.*, 2016	0–1000 nM for 6 h (cells) 25 μg/kg for 24 h (rats)	MCF-7 cells and adult, female Sprague–Dawley rats
Prins *et al.*, 2008	10 μg/kg body weight/day for 4 days	Sprague–Dawley rats
Chen *et al.*, 2021	10, 10^2^, 10^3^, 10^4^ and 10^5^ nM for 24, 48 and 72	TK6 and SUP-B15 cells
Zhang *et al.*, 2021	20 mg/kg body weight/day for 30 weeks	Sprague–Dawley rats
Weber Lozada *et al.*, 2011	0, 25 or 250 μg/kg until 8 weeks of age	FVB/N mice injected with MCF-7 cells
Jenkins *et al.*, 2011	0, 2.5, 25, 250 or 2500 μg/l from 56 until 112 or 252 days of age	MMTV-erbB2 mice
Prins *et al.*, 2014	100 or 250 μg/kg for 2 weeks	Nude mice
Jun *et al.*, 2021	0 to 100 μM from 0–5 days	HT-29 and CT-26 cells
Xia *et al.*, 2022	10^−7^, 10^−6^, 10^−5^, 10^−4^ and 10^−3^ mM from 0 to 24 h	SW620, HT-29, HCT116 and DLD1 cells
Weinhouse *et al.*, 2014	0, 50 ng, 50 μg or 50 mg/kg of diet for 10 months	*A^vy^*heterozygous mice
Sonavane *et al.*, 2018	150 μM BPA for 1 and 24 h	MEFs
Ribeiro *et al.*, 2019	4.4 μM (1 μg/ml), 4.4 nM (1 ng/ml) and 0.44 nM for 48 h	Hep-2 and MRC-5 cell lines
Kochmanski *et al.*, 2018	50 μg/kg diet for 2 weeks (maternal exposure)	*A^vy^*/*a* agouti mice

At the top (shaded in grey) the BPA doses found in human exposure studies are presented. Below (no shading) the doses and models used in experimental studies are presented.

Moreover, while the main focus of BPA on human health has revolved around metabolic and sexual dysfunction, recent literature has uncovered a role for BPA as a DNA damaging agent, mutagen, and potential carcinogen. For example, in animal models, *in utero* exposure to BPA has been shown to consistently alter mammary gland architecture (19–21). Moreover, perinatal exposure to BPA has been associated with an age dependent increase in mammary epithelial cell proliferation (22). While animal studies indicate a role for BPA as a potential carcinogen, reviewed in (23,24), many still yield conflicting results and the physiologic relevance of BPA concentrations remains a question.

This review aims to outline both mechanisms of BPA in promoting genomic instability as well as cellular proliferation and carcinogenesis and propose open questions and research directions in this critical field. An overview of the studies discussed, listing the concentration of BPA and the model system used, is presented in Table 1.

## Mechanisms of BPA-induced genomic instability

A characteristic of most cancers, genomic instability refers to the increased frequency of acquired mutations, structural rearrangements, and copy number alterations in the genome. Caused by dysfunctional maintenance of the genome or exposure to carcinogens, genomic instability is primarily categorized by defects in DNA repair, DNA replication, cell cycle control or chromosome segregation. When genomic integrity is impaired, the potential for carcinogenesis greatly increases, highlighting the importance of maintaining genomic stability (25).

While not currently classified as a carcinogen, BPA has been shown to damage DNA, leading to genomic instability and potentially carcinogenesis (26). Potential mechanisms through which this occurs, discussed in this review, include impairment of chromosomal segregation, DNA adduct formation, single and double stranded DNA break formation, reactive oxygen species formation, and estrogen receptor-mediated DNA damage.

### Impaired chromosome segregation

As previously stated, one of the mechanisms of promoting genomic instability is deregulation of chromosomal segregation. This is primarily maintained via the mitotic spindle assembly checkpoint (SAC), a surveillance mechanism that promotes proper segregation of chromosomes during anaphase. Impairment of this process leads to gains, losses, or rearrangement of genomic material with the potential to cause defects in birth and development and promote carcinogenesis (27).

Highlighting one of the ways in which BPA can promote genomic instability, a 2003 study revealed that exposure to BPA adversely affects chromosome segregation, promoting mitotic aneuploidy in female mice (10). After accidental exposure to BPA from damaged cages, it was found that exposure to low doses of BPA ranging from 20 to 100 ng/g disrupted meiosis of female mice in a dose dependent manner (10). Additionally, a study from 2010 further characterized the role of BPA in impairing chromosomal segregation, revealing that BPA exposure impedes chromosome synapsis and disrupts progression of meiotic double strand break repair (DSBR) in a *C. elegans* model (28). This study showed that exposure to BPA impairs oogenesis, resulting in elevated levels of sterility and embryonic lethality. Moreover, the ability of BPA to act as a xenoestrogen resulted in a germline specific down-regulation of DSBR genes such as MRE11, hindering the maintenance of genomic integrity during meiosis (28). While the effects of BPA on mitotic progression in the context of ER activation remain primarily unknown, it has also been shown that BPA-mediated ER activation promotes enhanced activation of the Src/Raf/Erk pathway, leading to chromosome malsegregation (29). Because ER activation promotes cellular proliferation, it is important to uncover whether BPA-mediated ER activation hinders mitotic progression or results in suppression of checkpoint control pathways, leading to further chromosomal instability.

In an attempt to further define the mechanisms in which BPA exposure leads to genomic instability and carcinogenesis, a study by Kim *et al.*, investigated the effects of BPA on mitosis progression, revealing that BPA interferes with the attachment of spindle microtubules to kinetochores (30). By disturbing spindle attachment and activating the SAC, BPA prolongs mitotic progression. Additionally, BPA perturbs the localization of microtubule associated proteins, HURP and TPX2. BPA also generates a multipolar spindle by inducing over duplication of the centriole and premature disengagement. This is shown to occur in an estrogen receptor (ER) independent manner (30).

### DNA damage and repair

When a DNA base is chemically modified, the damaged base must be repaired or excised in order to ensure faithful replication of the DNA and prevent mutagenesis. This can occur through processes such as direct reversal, base excision repair, or nucleotide excision repair in which the modified base is removed and subsequently replaced by a DNA polymerase. However, some adducts may escape DNA repair, and persist into S-phase (31).

When a cell's replication machinery encounters a DNA adduct, this can lead to replication stress, a major cause of genomic instability. These adducts can either be replicated through, via a mechanism known as translesion DNA synthesis (TLS), or the DNA synthesis downstream of the adduct can be reprimed by PrimPol, a polymerase with priming capability (32). Replicating over the base may lead to nucleotide misincorporation, in which a mutation of the base opposite the lesion or at the position corresponding to the lesion becomes fixed during the first and second round of replication, respectively. On the other hand, repriming will induce the formation of a single stranded DNA (ssDNA) gap, which when transmitted to the next cycle of DNA replication, leads to collapse of the replication fork, resulting in further genomic instability (32).

**Figure 1.**
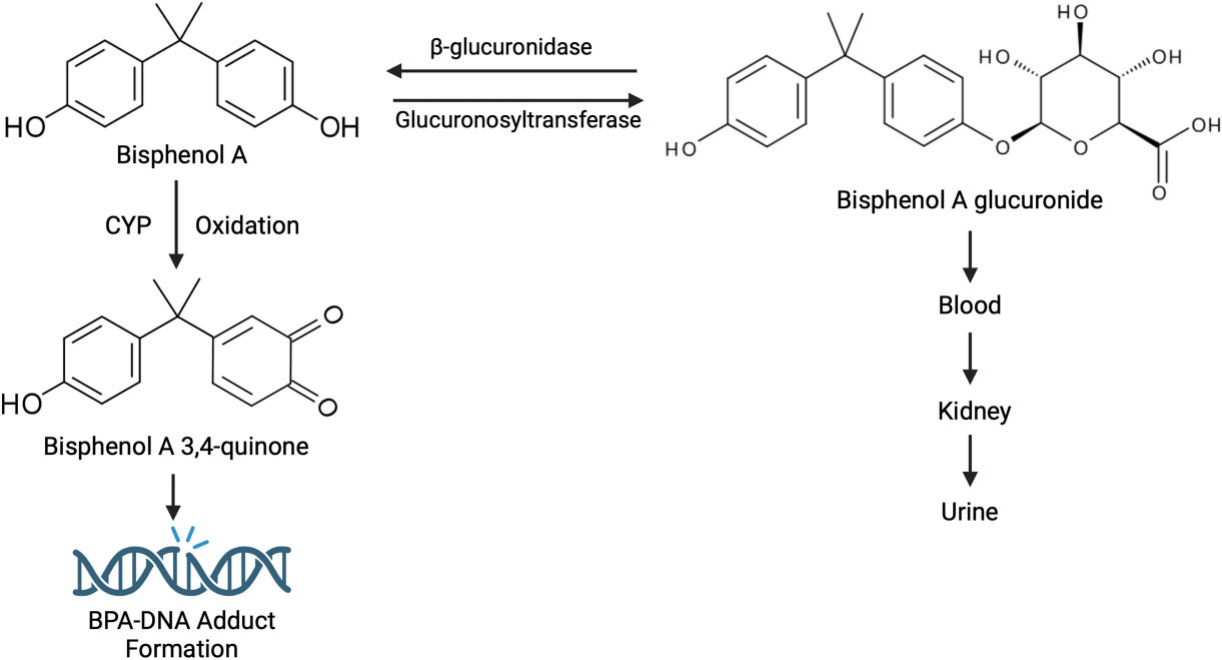
Structure and metabolization of BPA. After BPA enters the body, it is metabolized and inactivated in the liver by the addition of glucuronide, forming Bisphenol A glucuronide. This compound is then filtered out of the blood by the kidneys and is excreted in the urine. BPA can also be deconjugated and reactivated by β-glucuronidase. Unconjugated BPA is oxidized by cytochrome P450 (CYP), forming Bisphenol A 3,4-quinone, which has the potential to form adducts with DNA. Created in BioRender. Moldovan, G. (2024) BioRender.com/o52i455.

BPA has been shown to form adducts with DNA, specifically after oxidation into bisphenol-*o*-quinone (BPAQ) (33). Once BPA enters the body, it is metabolized and inactivated in the liver by the addition of glucuronide, forming Bisphenol A glucuronide. This conjugate has a half-life of 2 h and is primarily secreted into the urine (33). However, BPA can also be deconjugated and reactivated by β-glucuronidase, an enzyme found in tissues such as the placenta and the liver (34,35). This reactivated form of BPA is then able to form DNA adducts (Figure 1) (36).

BPA has been shown to form DNA adducts in human breast cancer cells as well as *in vivo* in murine liver and mammary tissue (37,38). A study in 2021 by Hu *et al.* investigated the effects of BPA adduct formation on the human genome, identifying patterns of genome-wide point mutations and genomic rearrangements associated with BPA exposure (39). Preferred sites of mutagenesis included regions at or near guanines, confirming the tendency of BPA to form guanine adducts. Moreover, BPA treatment resulted in a high frequency of insertions and deletions (InDels), DNA DSBs and structural variants, suggesting that BPA adducts result in DNA breaks during replication or are replicated through by translesion synthesis polymerases. Finally, it was shown that genomic signatures associated with BPA-mediated DNA damage exhibit high similarity to those of digestive and urinary tumors, both of which are associated with increased environmental exposure to BPA (39). While this study suggested that BPA adducts are replicated through by TLS polymerases, whether this occurs remains to be explored. Additionally, it is unknown which TLS polymerases engage at BPA-DNA lesions.

BPA has also been shown to promote the formation of single stranded and double stranded DNA breaks in replicating cells, presumably through its ability to form DNA adducts. In human hepatoma cell lines, BPA was shown to induce the formation of DNA breaks after 24 h, indicating that DNA replication must occur before breaks can be detected (40). Moreover, BPA has also been shown to promote a dose dependent increase in single and double stranded DNA breaks in human peripheral blood mononuclear cells (PBMCs) which were unable to be totally repaired (41).

Recently, our laboratory showed that BPA exposure causes the formation of nascent strand single stranded DNA (ssDNA) gaps which, in addition to homology directed repair (HDR), are fundamental to the sensitivity of BRCA deficient cells to genotoxic agents such as cisplatin and PARP inhibitors (42–45). When ssDNA accumulates, deposits of RPA, a protein that binds to and protects ssDNA, become depleted due to increased demand, and cells become increasingly reliant on post replicative mechanisms of DNA repair. The accumulation of ssDNA gaps leads to increased formation of dsDNA breaks, which are unable to be repaired in cells lacking efficient homologous recombination (HR) (46). Ultimately, the increase in DSB formation in these cells leads to genomic instability and cell death, potentially underlying the sensitivity of BRCA deficient cells to ssDNA gap inducing agents (47).

**Figure 2.**
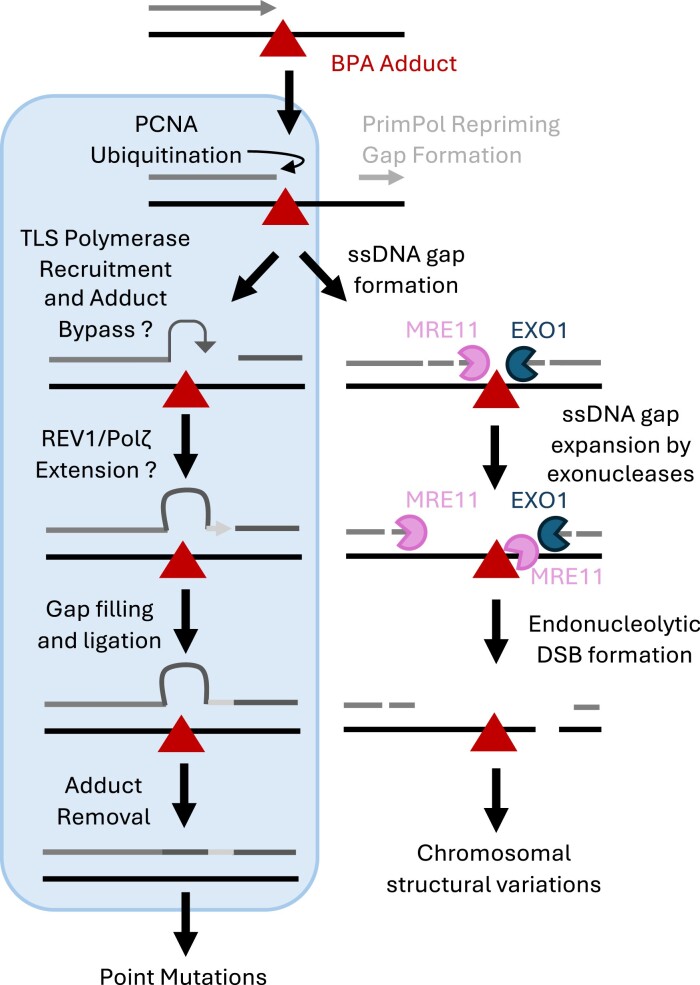
Proposed mechanisms of BPA-induced genomic instability during carcinogenesis. When the replication fork encounters a BPA adduct, repriming is initiated downstream of the lesion by PrimPol, leaving behind a ssDNA gap. This gap is then expanded bidirectionally by MRE11 and EXO1. If left unrepaired, the template strand opposite of the gap is nicked by the endonuclease activity of MRE11, forming a dsDNA break which can promote the accumulation of chromosomal aberrations. We hypothesize that BPA adducts can also be replicated through via TLS. TLS polymerases are capable of filling the gap, but they are also error prone, promoting the formation of point mutations.

Our recent study revealed that BPA promotes the accumulation of nascent strand ssDNA gaps (45). Moreover, we showed that these gaps are expanded bidirectionally by the MRE11 and EXO1 nucleases, and both expansion of the gap by the exonucleolytic activity of MRE11 and EXO1 as well as cleavage of the template strand by the endonucleolytic activity of MRE11 is required for conversion of the BPA induced ssDNA gap to a dsDNA break. Left unrepaired, the accumulation of DSBs will likely promote an increase in chromosomal structural variations, further increasing genomic instability (Figure 2). We also showed that exposure to BPA decreases cellular viability, which can be rescued by knockdown of EXO1, suggesting that exonucleolytic gap processing leads to cytotoxicity (45).

How BPA-mediated ssDNA gaps are repaired to avoid their conversion to DSBs is still unclear. As mentioned in the previous section, filling of these gaps or replication over BPA adducts may occur through TLS. While not yet shown, we hypothesize that formation of BPA adducts can also promote PCNA ubiquitination at stalled replication forks, an event which is required for TLS. This recruits TLS polymerases which can bypass the BPA adduct (Figure 2). Our model also implies that after the initial adduct bypass, REV1 and Polζ polymerases extend the DNA, resulting in gap filling and allowing ligation. Because TLS polymerases are error prone, this process may result in point mutations. As shown by Hu *et al.*, exposure to BPA results in both structural variants and point mutations, supporting the idea that these adducts can be replicated through by TLS or form ssDNA gaps which, when left unrepaired, result in endonucleolytic DSB formation (39,45).

### Reactive oxygen species formation

Formed as a byproduct of metabolism, reactive oxygen species (ROS) serve as a significant source of endogenous DNA damage, resulting in DNA base modifications, single- and double-strand breaks, and apurinic/apyrimidinic lesions (48). In turn, upon DNA damage, further induction of ROS is promoted via histone H2AX accumulation, shown to be mediated through Nox1 and the Rac1 GTPase (49,50).

Exposure to BPA has also been shown to promote the accumulation of ROS and enhance signaling of oxidative stress mediated signaling pathways (51). For example, BPA has been shown to decrease cellular viability via the depletion of the intracellular level of ATP and alterations in human peripheral blood mononuclear cell (PBMC) size and granulation (51). Even moderate reductions in levels of intracellular ATP are enough to promote oxidative stress (52). Moreover, BPA-mediated increases in ROS levels exhibit both damage to proteins and lipids in PBMCs (53). This increase in ROS formation promotes an increase in both single and double stranded DNA breaks as well as an increase in the expression of the DNA damage-associated proteins p53 and p-Chk2 (54). BPA has also been shown to induce oxidative stress by decreasing the antioxidant enzymes superoxide dismutase (SOD), catalase, glutathione reductase (GR) and glutathione peroxidase (GSH-Px) and increasing hydrogen peroxide and lipid peroxidation in the liver and epididymal sperm of rats (55).

While both BPA-driven adduct formation and ROS production can cause single and double stranded DNA breaks, the mutational signatures of the two have yet to be differentiated. Further avenues for investigation include distinguishing the mutational spectra of BPA-mediated DNA damage based on the mechanism in which it occurs. While it is possible that these signatures overlap, identifying mechanisms of BPA-mediated DNA damage may help to uncover if different routes of exposure cause damage in different ways. If so, this would greatly assist in guiding the development of mechanisms to minimize harm from BPA exposure. Additionally, whether repair of BPA adduct-derived ssDNA gaps occurs via the same mechanism as repair of damage from ROS is still unclear. Understanding which origins of damage promote which mechanism of DNA damage tolerance and repair is important to fully outline the effect of BPA on genomic instability.

### Estrogen receptor mediated DNA damage

Also important to consider is the role of BPA as a xenoestrogen and how its activity as an endocrine disruptor affects DNA damage and repair. Activation of the estrogen receptor (ER) has been shown to promote genomic instability. For example, estrogen (E2) mediated ER activation has been shown to enhance the formation of co-transcriptional R-loops, which have been linked to DNA damage (56). Activation of the ER has also been shown to increase the transcription of genes involved in the repair of double strand DNA breaks such as MRE11, RAD50 and PALB2, and ER-mediated transcription has also been shown to induce the formation of cell-cycle dependent dsDNA breaks (57,58). Intriguingly, since MRE11 also expands BPA-induced ssDNA gaps, increased MRE11 levels after ER activation may further promote gap-induced genomic instability. ER activation also promotes cellular proliferation through upregulation of protooncogenes and growth factors such as c-myc, and reliance on ER signaling is a critical regulator of cellular proliferation and survival in a majority of breast cancers (59).

In addition to the role of E2 in inducing DNA damage via ER activation, BPA has also been shown to promote genomic instability through its estrogenic activity. For example, BPA exposure promotes the formation of dsDNA breaks to a greater extent in ER-expressing cells than ER-negative cells (60). As previously mentioned, BPA-mediated ER activation promotes enhanced activation of the Src/Raf/Erk pathway, leading to chromosome mis-segregation and increased micronuclei formation (29).

## Additional mechanisms of BPA in cancer etiology and treatment

Despite scientific evidence suggesting that BPA promotes DNA damage and carcinogenesis, BPA is not classified as a carcinogen by the Environmental Protection Agency, primarily due to lack of consistency between studies (15,61). Moreover, as chemoresistance remains a key problem in the clinic, understanding of carcinogenic risk factors and how they relate to tumor formation remains essential to enhancing treatment decisions. The following section will focus on mechanisms in which BPA promotes aberrant cellular proliferation as well as evidence linking BPA exposure to chemotherapy resistance.

### Transcriptional effects

**Figure 3.**
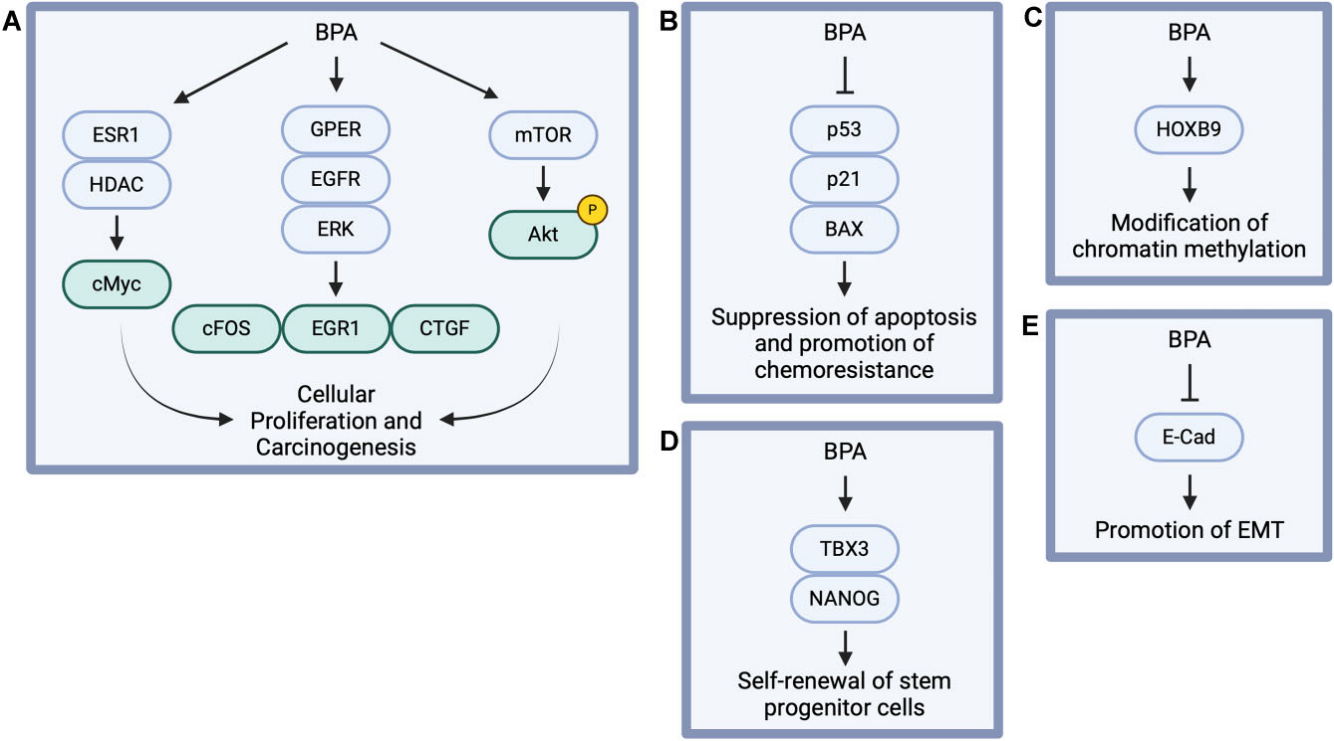
BPA may promote carcinogenesis through alteration of transcriptional activity. BPA has been shown to upregulate c-myc via ESR1 and HDAC activation. BPA also can activate the GPER/EGFR/ERK and mTOR signal transduction pathways, promoting cellular proliferation and carcinogenesis (**A**). BPA exposure also promotes reduced expression of proapoptotic regulators of the cell cycle including, p53, p21^WAF1^ and BAX, enhancing chemoresistance (**B**). BPA has been shown to induce the expression of HOXB9, a homeobox protein known to modify chromatin methylation and promote gene activation (**C**). BPA can also upregulate TBX3 and NANOG, enhancing the self-renewal of stem progenitor cells (**D**). Finally, BPA inhibits expression of E-Cadherin, promoting epithelial to mesenchymal transition, associated with metastasis (**E**). Created in BioRender. Moldovan, G. (2024) BioRender.com/s44z602.

One way in which BPA may exert a carcinogenic effect is through transcriptional regulation. For example, BPA exposure has been shown to upregulate the expression of c-MYC in estrogen receptor-α (ERα)-negative mammary cells, promoting DNA damage and cellular proliferation (62). This is thought to occur via induction of HDAC6 expression which, in turn, downregulates the tumor suppressor gene PTEN, resulting in upregulation of c-MYC expression (63). Silencing of c-myc reduces BPA-mediated DNA damage and cellular proliferation, suggesting that expression of c-MYC is essential for regulating effects of BPA on DNA damage and proliferation in mammary cells (62). BPA has also been shown to induce the expression of the GPER (G-protein coupled estrogen receptor) target genes c-FOS, EGR-1 and CTGF through the GPER/EGFR/ERK transduction pathway in SKBR3 breast cancer cells and cancer associated fibroblasts (CAFs) (64). Similar to c-MYC, the proliferative effects of BPA were reduced when GPER expression was silenced, indicating that BPA-mediated cellular proliferation relies on upregulation of both GPER and c-MYC (64) (Figure 3A).

Moreover, BPA has also been shown to induce activation of the mammalian target of rapamycin (mTOR) pathway (65). In both human high-risk donor breast epithelial cells (HRBEC) and T47D breast cancer cells, BPA exposure promotes increased expression of proliferation-initiating gene products such as proliferating cell nuclear antigen (PCNA), cyclins, CDKs and phosphorylated Rb (65) (Figure 3A). BPA exposure also promotes reduced expression of proapoptotic regulators of the cell cycle including, p53, p21^WAF1^ and BAX (Figure 3B). BPA was also shown to extend the proliferation potential of 6 independent HRBEC cultures (65). Continuous growth of one of the HRBEC cultures was even maintained after BPA removal, suggesting that BPA promotes genomic instability-driven hyperproliferation (65).

Additionally, BPA plays a role in the dysregulation of genes associated with physiological development and cancer pathogenesis. For example, BPA has been shown to induce the expression of HOXB9, a homeobox-containing gene that plays a key role in mammary gland development (66). While HOXB9 is transcriptionally regulated by estradiol (E2), the estrogen-response-elements (EREs) in the HOXB9 promoter can also be stimulated in the presence of BPA, modifying chromatin methylation, and promoting gene activation (66) (Figure 3C).

In addition to mammary development, BPA has also been shown to activate catenin-beta-1 (CTNNB1), a key gene indicated in lymphoma pathogenesis (67). Activation of CTNNB1 by BPA results in an increase in DNA SSB and DSB damage as well as cell cycle arrest in the G2/M phase for DNA repair (67).

### Cellular proliferation and tumorigenesis

In addition to promoting transcription of genes implicated in cancer pathogenesis, BPA exposure has also been directly associated with aberrant cellular proliferation and transformation. For example, BPA alone, or in combination with di(2- ethylhexyl) phthalate (DEHP) has been shown to induce mammary gland hyperplasia and promote proliferation of ductal epithelial cells, resulting in an increase in lobule and acini formation (68). BPA exposure also reduces tumor latency, enhancing the susceptibility of mammary tissue to carcinogenesis. These findings were associated with upregulation of ESR1 and HDAC6 which led to a further activation of c-MYC, also shown to be essential to BPA-induced mammary cell proliferation (62,68) (Figure 3A).

Moreover, fetal mice exposed to BPA have been shown to exhibit increased susceptibility to DMBA-induced tumor formation. BPA was also shown to promote established tumor growth in mice injected with MCF-7 human breast cancer cells which was reversed by tamoxifen treatment (69). These findings indicate BPA’s role in promoting cancer progression through both molecular alteration of fetal glands and estrogen receptor-dependent cell growth. Low doses of BPA corresponding to 25 μg BPA/l were also shown to promote mammary tumorigenesis and metastasis in MMTV-erbB2 mice (70). Decreased tumor latency and increased tumor burden in the presence of BPA was associated with increased phosphorylation of erbB2, erbB3, insulin-like growth factor 1 receptor, and Akt in the mammary gland (70).

BPA has also been shown to promote the self-renewal of stem-progenitor cells, specifically those in the human prostate. BPA exposure was found to induce phosphorylation of Akt and Erb as well as increase the expression of stem-related genes such as TBX3 and NANOG (71) (Figure 3D). Moreover, continuous exposure to BPA increased high-grade prostate intraepithelial neoplasia and adenocarcinoma in human prostate epithelial grafts composed of prostate stem-progenitor cells (71).

In addition, BPA has been shown to promote both the proliferation and migration of T-29 human colon adenocarcinoma cells via phosphorylation of the extracellular signal-regulated kinase (ERK) (72). BPA also reduced E-Cadherin expression, promoting epithelial to mesenchymal transition, and increased 5-HT3 receptor expression, a key mitogenic factor (72) (Figure 3E). Moreover, BPA exposure has been shown to promote excessive ROS production, subsequently activating the HIF-1α/VEGF/PI3K/AKT axis and promoting the growth and migration of human colon cancer cells (73). BPA has also been shown to promote a dose-dependent increase in hepatic tumor incidence in adult mice following perinatal exposure (74).

While this evidence suggests that BPA may function as a direct carcinogen, other studies have indicated that BPA may promote carcinogenesis via epigenetic modifications. For example, BPA has been shown to alter DNA methylation patterns in genes responsible for prostate development. After BPA exposure, areas of hypomethylation were identified in a 5′-flanking CpG island of the phosphodiesterase type 4 variant 4 (PDE4D4) gene (75). This region is typically gradually hypermethylated with age in the human prostate. However, hypomethylation and increased PDE4D4 expression is often observed in prostate cancer formation, indicating a role for BPA in prostate cancer pathogenesis (75). Additionally, a study by the Dolinoy group investigated the effects of perinatal BPA exposure on longitudinal 5-hmC patterns at imprinted regions of the genome, showing that differentially hydroxymethylated regions of the murine genome exhibit consistent patterns of differential 5-hmC by developmental BPA exposure that persisted throughout adulthood (76). Further understanding of the interplay between BPA’s role as a genotoxic agent and an epigenetic modifier is essential to fully outline its role in promoting carcinogenesis.

### Chemotherapy resistance

While great advancements have been made in the way cancer is treated, resistance to chemotherapy remains a key clinical problem (77). In cancer patients receiving traditional chemotherapeutics or novel targeted drugs, multidrug resistance is responsible for over 90% of patient deaths (78). While chemotherapeutic resistance is primarily thought to be due to genetic changes involving the agent itself, exposure to environmental agents such as BPA can also promote resistance to treatment. For example, co-exposure of BPA with camptothecin (CPT), a topoisomerase inhibitor, was found to decrease the burden of Top1–DNA adducts as well as chromosomal aberrations and DNA strand break formation in mouse embryonic fibroblasts (MEFs) (79). Increased cellular survival due to BPA exposure was associated with wide-spread compaction of chromatin and loss of nuclear volume, reducing the accessibility of DNA to Top1, and inhibiting the function of CPT (79).

Moreover, BPA has also been shown to interfere with the function of the anthracycline doxorubicin, a topoisomerase II inhibitor. BPA exposure upregulated the expression of bcl-xl, a critical anti-apoptotic protein and reduces the sensitivity of Hep-2 and MRC5 cells to doxorubicin (80). These studies suggest that, while BPA typically promotes genomic stability and aberrant cellular proliferation via mechanisms described above, it may also directly impact the mechanism of action of topoisomerase inhibitors. This indicates the importance of outlining the function of BPA as it relates to genomic instability so that informed treatment decisions can be made, specifically as related to populations with high exposure levels. Altogether, these findings indicate that BPA exposure plays a role in influencing the outcomes of chemotherapy.

## Future directions

Future directions of research could focus on identifying more accurate measurements of human exposure to BPA. Typically, human exposure is reported as levels of conjugated BPA in either urine or serum. However, studies have shown that biomonitoring of BPA via blood or urine may underestimate the total body burden. For example, BPA can also be excreted and detected in sweat and adipose tissue, even in individuals whose urine or serum samples exhibit no traces of BPA (11,81). Moreover, BPA has also been shown to have a disproportionate affinity for fat as compared to other tissues including kidney and muscle tissue (82). These findings indicate a need for further investigation of BPA accumulation in the human body as well as its potential to exert longitudinal effects on genomic stability. Additionally, given that BPA, like other estrogenic chemicals, exhibits a non-monotonic dose relationship, establishing whether higher levels of exposure for a shorter duration mimic the lifetime exposure in most humans is imperative to consider the relevance of findings obtained in cell-culture studies and how they may translate to human health.

Moreover, there is a need to further investigate the effect of BPA-mediated ER activation on promoting genomic instability. While it has been shown that BPA exerts a greater DNA damaging effect on cells expressing the estrogen receptor, little is known about the mechanism in which this occurs. Investigating BPA-mediated gene regulation via ER activation, particularly genes which play a role in exacerbating BPA-mediated DNA damage, such as MRE11 and EXO1, is necessary to further define this mechanism. Additionally, because ER activation also promotes cellular proliferation, it is important to uncover whether BPA mediated-ER activation plays a role in further hindering mitotic progression or results in suppression of checkpoint pathways, leading to further chromosomal instability. It would also be interesting to compare the role of BPA in cancer initiation in ER+ and ER– models, as BPA also seems to enhance cellular proliferation in a manner independent of ER activation.

Another aspect of note is further defining the mechanism by which BPA promotes ssDNA gap and dsDNA break formation, independent of ER activation. While it has been shown that BPA mutational signatures include point mutations as well as chromosomal aberrations, published work has mainly focused on the role of BPA in promoting nascent strand ssDNA gaps via PrimPol mediated repriming. It would also be interesting to investigate whether BPA lesions on DNA are able to be replicated through by TLS polymerases as well as the mechanism in which BPA-derived ssDNA gaps are filled to suppress their conversion into dsDNA breaks.

In addition, because BPA is able to form DNA adducts, it may exhibit a more pronounced genotoxic effect when cells are proliferating. Moreover, BPA exposure has also been shown to promote the proliferation of cells, potentially exacerbating this phenomenon. The intersection between increased cellular proliferation and accumulation of genotoxicity as it relates to BPA exposure has yet to be fully understood. Investigating the relationship between these variables in both ER-dependent and independent backgrounds is necessary to fully uncover the role of BPA in promoting genotoxicity and ultimately carcinogenesis.

Finally, future directions also include identifying genes that, when lost, predispose individuals to BPA-mediated DNA damage and cellular sensitivity. Methods to investigate this include CRISPR and shRNA screens. Identifying populations that are more vulnerable to BPA-mediated genomic instability could help establish safety guidelines that prevent these individuals from excess exposure to BPA, particularly those employed in the plastic manufacturing industry. Overall, further investigation of the effects of BPA on genomic instability is crucial to understand how this chemical plays a role in promoting carcinogenesis.

## Conclusion

While BPA exposure has been associated with a wide variety of adverse health effects such as abnormalities in reproductive organ function, placental dysfunction, precocious puberty, and neurological impairment, its role as a carcinogen is less defined. Via either formation of adducts with DNA or activation of estrogen receptor signaling, recent studies suggested that BPA promotes genomic instability at both point mutations and chromosome structure levels, as well as oxidative damage to lipids and proteins. Through transcriptional regulation, BPA also promotes cellular proliferation and migration, resulting in decreased tumor latency and increased tumor burden. Moreover, BPA’s ability to impact chromatin structure and promote the evasion of apoptosis also leads to an increase in resistance to chemotherapy, a key clinical problem. While more work must be done to uncover the potential role of BPA in cancer formation and define its function as a carcinogen, recent studies summarized here have shown that BPA exposure results in genomic instability, which is considered an enabling characteristic of carcinogenesis. Knowledge about the physiological effects of BPA on DNA damage and cancer formation can be used to better define the mechanisms that guide DNA damage tolerance as well as guide treatment decisions in patients affected by BPA exposure. Finally, identifying the mechanisms by which BPA causes DNA damage will help to define the health risks of BPA and serve to outline measures that may be taken to further decrease human exposure.

## Data Availability

No new data were generated or analysed in support of this research.

## References

[1] Center for Disease Control National Health and Nutrition Examination Survey 2003–2004 data documentation, codebook, and frequencies. 2013;

[2] Dodds E.C. The pharmacological action and clinical use of drugs with a camphor- and coramine-like action: (Section of Therapeutics and Pharmacology). Proc. R. Soc. Med. 1936; 29:655–657. 19990682 10.1177/003591573602900616 PMC2075731

[3] Thomas P., Dong J. Binding and activation of the seven-transmembrane estrogen receptor GPR30 by environmental estrogens: a potential novel mechanism of endocrine disruption. J. Steroid Biochem. Mol. Biol. 2006; 102:175–179. 17088055 10.1016/j.jsbmb.2006.09.017

[4] Colorado-Yohar S.M., Castillo-González A.C., Sánchez-Meca J., Rubio-Aparicio M., Sánchez-Rodríguez D., Salamanca-Fernández E., Ardanaz E., Amiano P., Fernández M.F., Mendiola J. et al. Concentrations of bisphenol-A in adults from the general population: a systematic review and meta-analysis. Sci. Total Environ. 2021; 775:145755. 34132197 10.1016/j.scitotenv.2021.145755

[5] Manzoor M.F., Tariq T., Fatima B., Sahar A., Tariq F., Munir S., Khan S., Ranjha M.M.A.N., Sameen A., Zeng X.A. et al. An insight into bisphenol A, food exposure and its adverse effects on health: a review. Front. Nutr. 2022; 9:1047827. 36407508 10.3389/fnut.2022.1047827 PMC9671506

[6] Calafat A.M., Weuve J., Ye X.Y., Jia L.T., Hu H., Ringer S., Huttner K., Hauser R. Exposure to bisphenol A and other phenols in neonatal Intensive Care unit premature infants. Environ. Health Perspect. 2009; 117:639–644. 19440505 10.1289/ehp.0800265 PMC2679610

[7] Kanno Y., Okada H., Kobayashi T., Takenaka T., Suzuki H. Effects of endocrine disrupting substance on estrogen receptor gene transcription in dialysis patients. Ther. Apher. Dial. 2007; 11:262–265. 17661831 10.1111/j.1744-9987.2007.00472.x

[8] Murakami K., Ohashi A., Hori H., Hibiya M., Shoji Y., Kunisaki M., Akita M., Yagi A., Sugiyama K., Shimozato S. et al. Accumulation of bisphenol A in hemodialysis patients. Blood. Purif. 2007; 25:290–294. 17622711 10.1159/000104869

[9] Hines C.J., Jackson M.V., Deddens J.A., Clark J.C., Ye X., Christianson A.L., Meadows J.W., Calafat A.M. Urinary bisphenol A (BPA) concentrations among workers in industries that manufacture and use BPA in the USA. Ann. Work Expo Health. 2017; 61:164–182. 28395354 10.1093/annweh/wxw021 PMC5577557

[10] Hunt P.A., Koehler K.E., Susiarjo M., Hodges C.A., Ilagan A., Voigt R.C., Thomas S., Thomas B.F., Hassold T.J. Bisphenol a exposure causes meiotic aneuploidy in the female mouse. Curr. Biol. 2003; 13:546–553. 12676084 10.1016/s0960-9822(03)00189-1

[11] Genuis S.J., Beesoon S., Birkholz D., Lobo R.A. Human excretion of bisphenol A: blood, urine, and sweat (BUS) study. J. Environ. Public Health. 2012; 2012:185731. 22253637 10.1155/2012/185731 PMC3255175

[12] National Institute of Environmental Health Sciences Bisphenol A (BPA). 2023; (27 April 2024, date last accessed) https://www.niehs.nih.gov/health/topics/agents/sya-bpa,.

[13] Xi W., Lee C.K., Yeung W.S., Giesy J.P., Wong M.H., Zhang X., Hecker M., Wong C.K. Effect of perinatal and postnatal bisphenol A exposure to the regulatory circuits at the hypothalamus-pituitary-gonadal axis of CD-1 mice. Reprod. Toxicol. 2011; 31:409–417. 21182934 10.1016/j.reprotox.2010.12.002

[14] US Environmental Protection Agency Bisphenol A Action Plan. 2010; (30 July 2024, date last accessed) https://www.epa.gov/sites/default/files/2015-09/documents/bpa_action_plan.pdf,.

[15] Prins G.S., Patisaul H.B., Belcher S.M., Vandenberg L.N. CLARITY-BPA academic laboratory studies identify consistent low-dose bisphenol A effects on multiple organ systems. Basic Clin. Pharmacol. Toxicol. 2019; 125(Suppl. 3):14–31. 30207065 10.1111/bcpt.13125 PMC6414289

[16] National Toxicology Program Carcinogenesis bioassay of bisphenol A (CAS No. 80-05-7) in F344 rats and B6C3F1 mice (Feed Study). Natl. Toxicol. Program Tech. Rep. Ser. 1982; 215:1–116. 12778220

[17] Vandenberg L.N., Colborn T., Hayes T.B., Heindel J.J., Jacobs D.R. Jr., Lee D.H., Shioda T., Soto A.M., vom Saal F.S., Welshons W.V. et al. Hormones and endocrine-disrupting chemicals: low-dose effects and nonmonotonic dose responses. Endocr. Rev. 2012; 33:378–455. 22419778 10.1210/er.2011-1050 PMC3365860

[18] Welshons W.V., Thayer K.A., Judy B.M., Taylor J.A., Curran E.M., vom Saal F.S. Large effects from small exposures. I. Mechanisms for endocrine-disrupting chemicals with estrogenic activity. Environ. Health Perspect. 2003; 111:994–1006. 12826473 10.1289/ehp.5494 PMC1241550

[19] Wang D., Gao H., Bandyopadhyay A., Wu A., Yeh I.T., Chen Y., Zou Y., Huang C., Walter C.A., Dong Q. et al. Pubertal bisphenol A exposure alters murine mammary stem cell function leading to early neoplasia in regenerated glands. Cancer Prev. Res. (Phila.). 2014; 7:445–455. 24520039 10.1158/1940-6207.CAPR-13-0260 PMC3976434

[20] Wadia P.R., Cabaton N.J., Borrero M.D., Rubin B.S., Sonnenschein C., Shioda T., Soto A.M. Low-dose BPA exposure alters the mesenchymal and epithelial transcriptomes of the mouse fetal mammary gland. PLoS One. 2013; 8:e63902. 23704952 10.1371/journal.pone.0063902 PMC3660582

[21] Betancourt A.M., Wang J., Jenkins S., Mobley J., Russo J., Lamartiniere C.A. Altered carcinogenesis and proteome in mammary glands of rats after prepubertal exposures to the hormonally active chemicals bisphenol a and genistein. J. Nutr. 2012; 142:1382S–1388S. 22649256 10.3945/jn.111.152058 PMC3374674

[22] Ayyanan A., Laribi O., Schuepbach-Mallepell S., Schrick C., Gutierrez M., Tanos T., Lefebvre G., Rougemont J., Yalcin-Ozuysal O., Brisken C. Perinatal exposure to bisphenol a increases adult mammary gland progesterone response and cell number. Mol. Endocrinol. 2011; 25:1915–1923. 21903720 10.1210/me.2011-1129 PMC5417179

[23] Seachrist D.D., Bonk K.W., Ho S.M., Prins G.S., Soto A.M., Keri R.A. A review of the carcinogenic potential of bisphenol A. Reprod. Toxicol. 2016; 59:167–182. 26493093 10.1016/j.reprotox.2015.09.006 PMC4783235

[24] Wang Z., Liu H., Liu S. Low-dose bisphenol A exposure: a seemingly instigating carcinogenic effect on breast cancer. Adv Sci (Weinh). 2017; 4:1600248. 28251049 10.1002/advs.201600248 PMC5323866

[25] Langie S.A., Koppen G., Desaulniers D., Al-Mulla F., Al-Temaimi R., Amedei A., Azqueta A., Bisson W.H., Brown D.G., Brunborg G. et al. Causes of genome instability: the effect of low dose chemical exposures in modern society. Carcinogenesis. 2015; 36(Suppl. 1):S61–S88. 26106144 10.1093/carcin/bgv031 PMC4565613

[26] Jalal N., Surendranath A.R., Pathak J.L., Yu S., Chung C.Y. Bisphenol A (BPA) the mighty and the mutagenic. Toxicol. Rep. 2018; 5:76–84. 29854579 10.1016/j.toxrep.2017.12.013 PMC5977157

[27] Curtis N.L., Ruda G.F., Brennan P., Bolanos-Garcia V.M. Deregulation of chromosome segregation and cancer. Annu. Rev. Cancer Biol. 2020; 4:257–278.

[28] Allard P., Colaiacovo M.P. Bisphenol A impairs the double-strand break repair machinery in the germline and causes chromosome abnormalities. Proc. Natl. Acad. Sci. U.S.A. 2010; 107:20405–20410. 21059909 10.1073/pnas.1010386107 PMC2996676

[29] Kabil A., Silva E., Kortenkamp A. Estrogens and genomic instability in human breast cancer cells–involvement of Src/Raf/erk signaling in micronucleus formation by estrogenic chemicals. Carcinogenesis. 2008; 29:1862–1868. 18544561 10.1093/carcin/bgn138

[30] Kim S., Gwon D., Kim J.A., Choi H., Jang C.Y. Bisphenol A disrupts mitotic progression via disturbing spindle attachment to kinetochore and centriole duplication in cancer cell lines. Toxicol. In Vitro. 2019; 59:115–125. 30980863 10.1016/j.tiv.2019.04.009

[31] Scharer O.D. Chemistry and biology of DNA repair. Angew. Chem. Int. Ed. Engl. 2003; 42:2946–2974. 12851945 10.1002/anie.200200523

[32] Quinet A., Tirman S., Cybulla E., Meroni A., Vindigni A. To skip or not to skip: choosing repriming to tolerate DNA damage. Mol. Cell. 2021; 81:649–658. 33515486 10.1016/j.molcel.2021.01.012 PMC7935405

[33] Volkel W., Colnot T., Csanady G.A., Filser J.G., Dekant W. Metabolism and kinetics of bisphenol a in humans at low doses following oral administration. Chem. Res. Toxicol. 2002; 15:1281–1287. 12387626 10.1021/tx025548t

[34] Ginsberg G., Rice D.C. Does rapid metabolism ensure negligible risk from bisphenol A?. Environ. Health Perspect. 2009; 117:1639–1643. 20049111 10.1289/ehp.0901010 PMC2801165

[35] Basinska A., Florianczyk B. Beta-glucuronidase in physiology and disease. Ann. Univ. Mariae Curie Sklodowska Med. 2003; 58:386–389. 15323223

[36] Atkinson A., Roy D. In vivo DNA adduct formation by bisphenol A. Environ. Mol. Mutagen. 1995; 26:60–66. 7641708 10.1002/em.2850260109

[37] Zhao H., Wei J., Xiang L., Cai Z. Mass spectrometry investigation of DNA adduct formation from bisphenol A quinone metabolite and MCF-7 cell DNA. Talanta. 2018; 182:583–589. 29501196 10.1016/j.talanta.2018.02.037

[38] Izzotti A., Kanitz S., D’Agostini F., Camoirano A., De Flora S. Formation of adducts by bisphenol A, an endocrine disruptor, in DNA in vitro and in liver and mammary tissue of mice. Mutat. Res. 2009; 679:28–32. 19660573 10.1016/j.mrgentox.2009.07.011

[39] Hu X., Biswas A., Sharma A., Sarkodie H., Tran I., Pal I., De S. Mutational signatures associated with exposure to carcinogenic microplastic compounds bisphenol A and styrene oxide. NAR Cancer. 2021; 3:zcab004. 33718875 10.1093/narcan/zcab004 PMC7936647

[40] Fic A., Zegura B., Sollner Dolenc M., Filipic M., Peterlin Masic L. Mutagenicity and DNA damage of bisphenol A and its structural analogues in HepG2 cells. Arh. Hig. Rada. Toksikol. 2013; 64:189–200. 23819927 10.2478/10004-1254-64-2013-2319

[41] Mokra K., Kuzminska-Surowaniec A., Wozniak K., Michalowicz J. Evaluation of DNA-damaging potential of bisphenol A and its selected analogs in human peripheral blood mononuclear cells (in vitro study). Food Chem. Toxicol. 2017; 100:62–69. 27923681 10.1016/j.fct.2016.12.003

[42] Cong K., Cantor S.B. Exploiting replication gaps for cancer therapy. Mol. Cell. 2022; 82:2363–2369. 35568026 10.1016/j.molcel.2022.04.023 PMC9271608

[43] Panzarino N.J., Krais J.J., Cong K., Peng M., Mosqueda M., Nayak S.U., Bond S.M., Calvo J.A., Doshi M.B., Bere M. et al. Replication gaps underlie BRCA deficiency and therapy response. Cancer Res. 2021; 81:1388–1397. 33184108 10.1158/0008-5472.CAN-20-1602 PMC8026497

[44] Lim P.X., Zaman M., Feng W., Jasin M. BRCA2 promotes genomic integrity and therapy resistance primarily through its role in homology-directed repair. Mol. Cell. 2024; 84:447–462. 38244544 10.1016/j.molcel.2023.12.025 PMC11188060

[45] Hale A., Dhoonmoon A., Straka J., Nicolae C.M., Moldovan G.L. Multi-step processing of replication stress-derived nascent strand DNA gaps by MRE11 and EXO1 nucleases. Nat. Commun. 2023; 14:6265. 37805499 10.1038/s41467-023-42011-0 PMC10560291

[46] Jackson L.M., Moldovan G.L. Mechanisms of PARP1 inhibitor resistance and their implications for cancer treatment. NAR Cancer. 2022; 4:zcac042. 36568963 10.1093/narcan/zcac042 PMC9773381

[47] Cong K., Peng M., Kousholt A.N., Lee W.T.C., Lee S., Nayak S., Krais J., VanderVere-Carozza P.S., Pawelczak K.S., Calvo J. et al. Replication gaps are a key determinant of PARP inhibitor synthetic lethality with BRCA deficiency. Mol. Cell. 2021; 81:3128–3144. 34216544 10.1016/j.molcel.2021.06.011 PMC9089372

[48] Salmon T.B., Evert B.A., Song B., Doetsch P.W. Biological consequences of oxidative stress-induced DNA damage in Saccharomyces cerevisiae. Nucleic Acids Res. 2004; 32:3712–3723. 15254273 10.1093/nar/gkh696 PMC484183

[49] Rowe L.A., Degtyareva N., Doetsch P.W. DNA damage-induced reactive oxygen species (ROS) stress response in Saccharomyces cerevisiae. Free Radic. Biol. Med. 2008; 45:1167–1177. 18708137 10.1016/j.freeradbiomed.2008.07.018 PMC2643028

[50] Kang M.A., So E.Y., Simons A.L., Spitz D.R., Ouchi T. DNA damage induces reactive oxygen species generation through the H2AX-Nox1/Rac1 pathway. Cell Death. Dis. 2012; 3:e249. 22237206 10.1038/cddis.2011.134 PMC3270268

[51] Wang K., Zhao Z., Ji W. Bisphenol A induces apoptosis, oxidative stress and inflammatory response in colon and liver of mice in a mitochondria-dependent manner. Biomed. Pharmacother. 2019; 117:109182. 31387175 10.1016/j.biopha.2019.109182

[52] Schutt F., Aretz S., Auffarth G.U., Kopitz J. Moderately reduced ATP levels promote oxidative stress and debilitate autophagic and phagocytic capacities in human RPE cells. Invest. Ophthalmol. Vis. Sci. 2012; 53:5354–5361. 22789922 10.1167/iovs.12-9845

[53] Michalowicz J., Mokra K., Bak A. Bisphenol A and its analogs induce morphological and biochemical alterations in human peripheral blood mononuclear cells (in vitro study). Toxicol. In Vitro. 2015; 29:1464–1472. 26028149 10.1016/j.tiv.2015.05.012

[54] Xin F., Jiang L., Liu X., Geng C., Wang W., Zhong L., Yang G., Chen M. Bisphenol A induces oxidative stress-associated DNA damage in INS-1 cells. Mutat. Res. Genet. Toxicol. Environ. Mutagen. 2014; 769:29–33. 25344109 10.1016/j.mrgentox.2014.04.019

[55] Bindhumol V., Chitra K.C., Mathur P.P. Bisphenol A induces reactive oxygen species generation in the liver of male rats. Toxicology. 2003; 188:117–124. 12767684 10.1016/s0300-483x(03)00056-8

[56] Stork C.T., Bocek M., Crossley M.P., Sollier J., Sanz L.A., Chedin F., Swigut T., Cimprich K.A. Co-transcriptional R-loops are the main cause of estrogen-induced DNA damage. eLife. 2016; 5:e17548. 27552054 10.7554/eLife.17548 PMC5030092

[57] Zach L.-o., Yedidia-Aryeh L., Goldberg M. Estrogen and DNA damage modulate mRNA levels of genes involved in homologous recombination repair in estrogen-deprived cells. J. Transl. Genet. Genom. 2022; 6:266–280.

[58] Williamson L.M., Lees-Miller S.P. Estrogen receptor alpha-mediated transcription induces cell cycle-dependent DNA double-strand breaks. Carcinogenesis. 2011; 32:279–285. 21112959 10.1093/carcin/bgq255

[59] Ciocca D.R., Fanelli M.A. Estrogen receptors and cell proliferation in breast cancer. Trends Endocrinol. Metab. 1997; 8:313–321. 18406820 10.1016/s1043-2760(97)00122-7

[60] Iso T., Watanabe T., Iwamoto T., Shimamoto A., Furuichi Y. DNA damage caused by bisphenol A and estradiol through estrogenic activity. Biol. Pharm. Bull. 2006; 29:206–210. 16462019 10.1248/bpb.29.206

[61] US Environmental Protection Agency Risk Management for Bisphenol A (BPA). 2023; (27 April 2024, date last accessed) https://www.epa.gov/assessing-and-managing-chemicals-under-tsca/risk-management-bisphenol-bpa,.

[62] Pfeifer D., Chung Y.M., Hu M.C. Effects of low-dose bisphenol A on DNA damage and proliferation of breast cells: the role of c-myc. Environ. Health Perspect. 2015; 123:1271–1279. 25933419 10.1289/ehp.1409199 PMC4671234

[63] Zhang X., Guo N., Jin H., Liu R., Zhang Z., Cheng C., Fan Z., Zhang G., Xiao M., Wu S. et al. Bisphenol A drives di(2-ethylhexyl) phthalate promoting thyroid tumorigenesis via regulating HDAC6/PTEN and c-MYC signaling. J. Hazard. Mater. 2022; 425:127911. 34910997 10.1016/j.jhazmat.2021.127911

[64] Pupo M., Pisano A., Lappano R., Santolla M.F., De Francesco E.M., Abonante S., Rosano C., Maggiolini M. Bisphenol A induces gene expression changes and proliferative effects through GPER in breast cancer cells and cancer-associated fibroblasts. Environ. Health Perspect. 2012; 120:1177–1182. 22552965 10.1289/ehp.1104526 PMC3440081

[65] Dairkee S.H., Luciani-Torres M.G., Moore D.H., Goodson W.H. 3rd Bisphenol-A-induced inactivation of the p53 axis underlying deregulation of proliferation kinetics, and cell death in non-malignant human breast epithelial cells. Carcinogenesis. 2013; 34:703–712. 23222814 10.1093/carcin/bgs379 PMC3581603

[66] Deb P., Bhan A., Hussain I., Ansari K.I., Bobzean S.A., Pandita T.K., Perrotti L.I., Mandal S.S. Endocrine disrupting chemical, bisphenol-A, induces breast cancer associated gene HOXB9 expression in vitro and in vivo. Gene. 2016; 590:234–243. 27182052 10.1016/j.gene.2016.05.009 PMC4975976

[67] Chen Y.K., Tan Y.Y., Yao M., Lin H.C., Tsai M.H., Li Y.Y., Hsu Y.J., Huang T.T., Chang C.W., Cheng C.M. et al. Bisphenol A-induced DNA damages promote to lymphoma progression in human lymphoblastoid cells through aberrant CTNNB1 signaling pathway. iScience. 2021; 24:102888. 34401669 10.1016/j.isci.2021.102888 PMC8350018

[68] Zhang X., Cheng C., Zhang G., Xiao M., Li L., Wu S., Lu X. Co-exposure to BPA and DEHP enhances susceptibility of mammary tumors via up-regulating Esr1/HDAC6 pathway in female rats. Ecotoxicol. Environ. Saf. 2021; 221:112453. 34186418 10.1016/j.ecoenv.2021.112453

[69] Weber Lozada K., Keri R.A. Bisphenol A increases mammary cancer risk in two distinct mouse models of breast cancer. Biol. Reprod. 2011; 85:490–497. 21636739 10.1095/biolreprod.110.090431 PMC3159535

[70] Jenkins S., Wang J., Eltoum I., Desmond R., Lamartiniere C.A. Chronic oral exposure to bisphenol A results in a nonmonotonic dose response in mammary carcinogenesis and metastasis in MMTV-erbB2 mice. Environ. Health Perspect. 2011; 119:1604–1609. 21988766 10.1289/ehp.1103850 PMC3226508

[71] Prins G.S., Hu W.Y., Shi G.B., Hu D.P., Majumdar S., Li G., Huang K., Nelles J.L., Ho S.M., Walker C.L. et al. Bisphenol A promotes human prostate stem-progenitor cell self-renewal and increases in vivo carcinogenesis in human prostate epithelium. Endocrinology. 2014; 155:805–817. 24424067 10.1210/en.2013-1955 PMC3929731

[72] Jun J.H., Oh J.E., Shim J.K., Kwak Y.L., Cho J.S. Effects of bisphenol A on the proliferation, migration, and tumor growth of colon cancer cells: in vitro and in vivo evaluation with mechanistic insights related to ERK and 5-HT3. Food Chem. Toxicol. 2021; 158:112662. 34743013 10.1016/j.fct.2021.112662

[73] Xia T., Guo J., Zhang B., Song C., Zhao Q., Cui B., Liu Y. Bisphenol A promotes the progression of colon cancer through dual-targeting of NADPH oxidase and mitochondrial electron-transport chain to produce ROS and activating HIF-1alpha/VEGF/PI3K/AKT axis. Front. Endocrinol. (Lausanne). 2022; 13:933051. 35860704 10.3389/fendo.2022.933051 PMC9289207

[74] Weinhouse C., Anderson O.S., Bergin I.L., Vandenbergh D.J., Gyekis J.P., Dingman M.A., Yang J., Dolinoy D.C. Dose-dependent incidence of hepatic tumors in adult mice following perinatal exposure to bisphenol A. Environ. Health Perspect. 2014; 122:485–491. 24487385 10.1289/ehp.1307449 PMC4014767

[75] Prins G.S., Tang W.Y., Belmonte J., Ho S.M. Perinatal exposure to oestradiol and bisphenol A alters the prostate epigenome and increases susceptibility to carcinogenesis. Basic Clin. Pharmacol. Toxicol. 2008; 102:134–138. 18226066 10.1111/j.1742-7843.2007.00166.x PMC2819392

[76] Kochmanski J.J., Marchlewicz E.H., Cavalcante R.G., Perera B.P.U., Sartor M.A., Dolinoy D.C. Longitudinal effects of developmental bisphenol A exposure on epigenome-wide DNA hydroxymethylation at imprinted loci in mouse blood. Environ. Health Perspect. 2018; 126:077006. 30044229 10.1289/EHP3441 PMC6108846

[77] Emran T.B., Shahriar A., Mahmud A.R., Rahman T., Abir M.H., Siddiquee M.F., Ahmed H., Rahman N., Nainu F., Wahyudin E. et al. Multidrug resistance in cancer: understanding molecular mechanisms, immunoprevention and therapeutic approaches. Front. Oncol. 2022; 12:891652. 35814435 10.3389/fonc.2022.891652 PMC9262248

[78] Bukowski K., Kciuk M., Kontek R. Mechanisms of multidrug resistance in cancer chemotherapy. Int. J. Mol. Sci. 2020; 21:3233. 32370233 10.3390/ijms21093233 PMC7247559

[79] Sonavane M., Sykora P., Andrews J.F., Sobol R.W., Gassman N.R. Camptothecin efficacy to poison Top1 is altered by bisphenol A in mouse embryonic fibroblasts. Chem. Res. Toxicol. 2018; 31:510–519. 29799191 10.1021/acs.chemrestox.8b00050 PMC6374779

[80] Ribeiro E., Delgadinho M., Brito M. Environmentally relevant concentrations of bisphenol A interact with doxorubicin transcriptional effects in Human cell lines. Toxics. 2019; 7:43. 31470548 10.3390/toxics7030043 PMC6789468

[81] Fernandez M.F., Arrebola J.P., Taoufiki J., Navalon A., Ballesteros O., Pulgar R., Vilchez J.L., Olea N. Bisphenol-A and chlorinated derivatives in adipose tissue of women. Reprod. Toxicol. 2007; 24:259–264. 17689919 10.1016/j.reprotox.2007.06.007

[82] Csanady G.A., Oberste-Frielinghaus H.R., Semder B., Baur C., Schneider K.T., Filser J.G. Distribution and unspecific protein binding of the xenoestrogens bisphenol A and daidzein. Arch. Toxicol. 2002; 76:299–305. 12107647 10.1007/s00204-002-0339-5

